# Projected Impact of Predefined Reflex Testing Algorithms on Thyroid Function and Prostate-Specific Antigen Testing: A Real-World Laboratory Stewardship Study

**DOI:** 10.3390/jcm15208050

**Published:** 2026-10-16

**Authors:** Murat Alisik, Merve Ates, Mehmet Alp Sert, Utku Gorkem Erdogan, Seda Takim Karaca, Mesut Ates, Ozgur Mehmet Yis

**Affiliations:** 1 Department of Medical Biochemistry, Faculty of Medicine, Bolu Abant İzzet Baysal University, 14030 Bolu, Türkiye; mervekaracats@gmail.com (M.A.); mehmet.alp3714@gmail.com (M.A.S.); utkuedogan@gmail.com (U.G.E.); sedatak94.dr@gmail.com (S.T.K.); dromyis@gmail.com (O.M.Y.); 2 Department of Internal Medicine, Faculty of Medicine, Bolu Abant İzzet Baysal University, 14030 Bolu, Türkiye; mesutatesdr@gmail.com

**Keywords:** laboratory stewardship, reflex testing, thyroid function testing, TSH, prostate-specific antigen, free PSA, test utilization, healthcare expenditure

## Abstract

**Background/Objectives**: Reflex testing may improve laboratory stewardship by limiting downstream analyses to situations in which additional testing is supported by the result of an initial test. This study evaluated the projected impact of predefined reflex testing algorithms on thyroid function and prostate-specific antigen (PSA) test utilization and laboratory expenditure. **Methods:** This observational modelling study included clinician-initiated thyroid function and PSA testing sessions recorded at a tertiary-care hospital in Türkiye during 2022. Clinician-requested tests and corresponding laboratory results were compared with predefined reflex algorithms applied retrospectively at the testing-session level. For thyroid testing, thyroid-stimulating hormone (TSH) was used as the initial test; free thyroxine (FT4) was projected for elevated TSH, and both FT4 and free triiodothyronine (FT3) for decreased TSH. For PSA testing, free PSA was projected only when total PSA was 4.0–10.0 ng/mL. Observed clinician-initiated testing was compared descriptively with projected algorithm-based testing, including downstream test utilization and laboratory expenditure. **Results:** The thyroid cohort comprised 60,656 testing sessions from 43,839 patients, and the PSA cohort comprised 4583 sessions from 3631 patients. In the thyroid cohort, clinicians requested 49,858 FT4 and 19,899 FT3 measurements. Under the predefined algorithm, these numbers were projected to decrease to 6205 and 2478, respectively, reducing total thyroid test volume from 130,413 to 69,339 tests, a projected reduction of 61,074 tests (46.8%). In the PSA cohort, free PSA testing was projected to decrease from 2133 to 642 measurements, reducing total PSA-related test volume from 6716 to 5225 tests (22.2%). Projected laboratory expenditure decreased from US dollars (USD) 73,579.01 to USD 39,121.06 for thyroid testing and from USD 6468.78 to USD 4560.73 for PSA testing. Across both pathways, the projected saving was USD 36,366.00, corresponding to a 45.4% reduction in expenditure. **Conclusions:** Retrospective application of predefined reflex testing algorithms to clinician-initiated thyroid function and PSA requests projected substantial reductions in downstream test utilization and laboratory expenditure. Prospective implementation studies incorporating patient-specific clinical information are needed to determine whether these projected reductions can be achieved safely in routine clinical practice without limiting clinically appropriate testing.

## 1. Introduction

Laboratory testing plays a central role in modern clinical decision-making, yet inappropriate test utilization remains an important challenge because both overuse and underuse may contribute to diagnostic errors, inefficient resource utilization, and unnecessary healthcare expenditure [1]. Laboratory stewardship seeks to optimize the quality, efficiency, and value of laboratory testing through interventions that improve test selection, ordering, and interpretation [2]. Among the available stewardship strategies, reflex testing and clinical decision support are particularly applicable to sequential diagnostic pathways in which the results of an initial test determine whether additional downstream analyses are required [3]. Thyroid function testing and prostate-specific antigen (PSA) testing are representative examples of such laboratory pathways and therefore provide suitable models for evaluating algorithm-based laboratory stewardship interventions.

Thyroid function testing represents one of the most frequently requested laboratory investigations in clinical practice. Because serum thyroid-stimulating hormone (TSH) is the most sensitive first-line marker of primary thyroid dysfunction, several professional organizations recommend a stepwise testing strategy in which additional thyroid hormone measurements are performed only when indicated by the initial TSH result [4,5]. For example, the 2023 European Thyroid Association guideline for thyroid nodule management recommends measuring free thyroxine (FT4) when TSH is decreased and proceeding to free triiodothyronine (FT3) if FT4 is normal, whereas elevated TSH should prompt FT4 measurement to further characterize thyroid dysfunction [4]. Similarly, the National Institute for Health and Care Excellence (NICE) guideline recommends a cascade approach in which subsequent thyroid hormone measurements are performed only after an abnormal TSH result, thereby avoiding unnecessary simultaneous testing while allowing additional analyses to be performed on the original specimen [5]. Despite these recommendations, concurrent ordering of TSH with FT4 and FT3 remains common in routine clinical practice, providing an opportunity to evaluate the potential impact of sequential algorithm-based testing [6,7]. Importantly, such sequential testing strategies are intended to guide routine test ordering rather than replace clinical judgment, as additional thyroid hormone measurements may be appropriate in selected clinical circumstances.

A similar sequential testing principle applies to PSA testing. Total PSA is recommended as the initial laboratory test, whereas free PSA is primarily intended to refine prostate cancer risk assessment when total PSA falls within an intermediate or laboratory-defined interpretive range [8]. Free PSA results outside this range cannot be interpreted with the same reliability, and the College of American Pathologists therefore recommends establishing the total PSA concentration before free PSA is measured or reported [8]. Reflex strategies in which free PSA is tested only when total PSA falls within the laboratory-defined range have consequently been proposed to improve test utilization [8]. Nevertheless, free PSA is frequently requested outside these conditions; in a large national reference-laboratory study, 38% of free PSA tests were performed when total PSA was below 2.5 ng/mL or above 10 ng/mL [9]. Contemporary discussions have also emphasized that percent free PSA should be interpreted in the context of total PSA rather than as an isolated estimate of prostate cancer risk [10]. These observations provide a rationale for evaluating sequential laboratory testing strategies in the PSA pathway alongside thyroid function testing.

Although thyroid function testing and PSA testing represent clinically distinct diagnostic pathways, both provide practical examples of sequential laboratory testing in which the result of an initial marker test can guide the need for downstream assays. They were therefore evaluated as two separate laboratory stewardship pathways rather than as clinically related conditions. The aim of this study was to retrospectively apply predefined reflex testing algorithms to these two pathways and estimate their pathway-specific effects on downstream test utilization and laboratory expenditure. The economic analysis was restricted to these selected pathways and was not intended to represent the overall expenditure of the laboratory or the overall economic impact of reflex testing across all laboratory services.

## 2. Materials and Methods

### 2.1. Study Design and Data Source

This observational study was conducted using laboratory test requests recorded at İzzet Baysal Education and Research Hospital, a tertiary-care university-affiliated hospital in Türkiye. Thyroid function tests and prostate-specific antigen (PSA) tests requested between 1 January and 31 December 2022 were extracted from the laboratory information system. All analyses were performed at the testing-session level. The study protocol was approved by the Bolu Abant İzzet Baysal University Clinical Research Ethics Committee (approval number: 2023/238), and the requirement for informed consent was waived because only de-identified laboratory data were analyzed.

### 2.2. Study Cohorts and Data Collection

Two laboratory testing pathways were evaluated independently: thyroid function testing and PSA testing. For each testing session, patient age, care setting (outpatient or inpatient), clinician-requested laboratory tests, and corresponding laboratory results were extracted from the hospital information management system. Only clinician-initiated test requests and their associated results were included in the analysis.

### 2.3. Predefined Testing Algorithms

Predefined reflex testing algorithms were developed before data analysis based on current guideline recommendations and laboratory stewardship principles. For each clinician-initiated testing session, the initial laboratory request was compared with the testing sequence that would have been generated by the predefined algorithm. The predefined algorithm was not implemented in routine clinical practice; rather, it was applied retrospectively to clinician-initiated requests to estimate its potential impact on downstream testing and laboratory expenditure.

For thyroid function testing, serum TSH was considered the initial test. If TSH was within the reference interval, no additional thyroid hormone measurements were projected. If TSH was above the reference interval, FT4 measurement was projected. If TSH was below the reference interval, both FT4 and FT3 measurements were projected from the same specimen. This predefined algorithm was established before data analysis to reflect a practical sequential testing strategy applicable to routine laboratory workflows and was informed by current guideline recommendations and laboratory stewardship principles.

For PSA testing, total PSA was considered the initial test. Free PSA measurement was projected only when the total PSA concentration was between 4.0 and 10.0 ng/mL. The 4.0–10.0 ng/mL interval was selected a priori as the predefined range for reflex free PSA testing in this study.

### 2.4. Outcome Measures

For each testing session, the clinician-initiated laboratory request was compared with the testing sequence projected by the predefined algorithm. The primary outcomes were the projected numbers of downstream laboratory tests and the projected laboratory expenditure under the predefined algorithms. Secondary outcomes included the numbers of clinician-initiated downstream tests classified as algorithm-defined potential overuse according to the predefined algorithms, the numbers of projected additional tests generated by the algorithms, and the projected changes in total test utilization. For the purposes of this analysis, clinician-initiated downstream tests that would not have been generated by the corresponding predefined algorithm were classified as algorithm-defined potential overuse. This classification reflects discordance with the predefined laboratory pathway and should not be interpreted as evidence that an individual test was clinically unnecessary, because patient-specific clinical indications were not available for assessment.

### 2.5. Cost Analysis

Laboratory test costs were calculated using the unit points listed in the Social Security Institution (SGK) of the Republic of Türkiye Fee-for-Service Procedure Point List (Annex 2/B), as amended on June 29, 2026 (Official Gazette No. 33295). Laboratory-specific reimbursement values were converted to Turkish Lira (TRY) using the applicable coefficient and subsequently converted to US dollars (USD) using an exchange rate of TRY 48.25 per USD. Projected laboratory expenditure was calculated by multiplying the projected number of tests generated by the predefined algorithms by the corresponding unit costs.

### 2.6. Statistical Analysis

Continuous variables are presented as median (interquartile range, IQR), and categorical variables as number (percentage). Descriptive analyses were performed separately for the thyroid function testing and PSA cohorts. Projected test utilization and laboratory expenditure under the predefined algorithms were compared descriptively with observed clinician-initiated testing. Statistical analyses were performed using JASP version 0.97.1 (JASP Team, Amsterdam, The Netherlands).

## 3. Results

### 3.1. Study Population

The thyroid function testing cohort comprised 60,656 testing sessions from 43,839 patients, and the PSA cohort comprised 4583 testing sessions from 3631 patients. Median age was 44.0 years (IQR, 25.0–61.0) in the thyroid cohort and 66.0 years (IQR, 58.0–72.0) in the PSA cohort. Cohort characteristics are presented in Table 1.

### 3.2. Clinician Ordering Patterns and Test Utilization

Initial clinician ordering patterns for thyroid function tests are summarized in Table 2. Of the 60,656 testing sessions, 30,001 (49.5%) included concurrent requests for TSH and FT4, while 19,857 (32.7%) included TSH, FT4, and FT3. In contrast, TSH alone was requested in 10,756 (17.7%) sessions, whereas requests for TSH and FT3 without FT4 were uncommon (42 sessions, 0.1%). Overall, 82.3% of clinician-initiated thyroid test requests included at least one downstream thyroid hormone measurement at the time of the initial order (Table 2).

Initial clinician ordering patterns for PSA testing are also shown in Table 2. Of the 4583 testing sessions, clinicians requested total PSA alone in 2450 (53.5%) sessions and concurrent total PSA and free PSA in 2133 (46.5%) sessions.

### 3.3. Projected Test Utilization Under the Predefined Algorithms

The distribution of TSH and total PSA results, together with the projected impact of applying the predefined testing algorithms, is summarized in Table 3. In the thyroid cohort, TSH concentrations were within the reference interval in 54,451 (89.8%) sessions, below the reference interval in 2478 (4.1%), and above the reference interval in 3727 (6.1%).

Under the predefined testing algorithm, the projected number of FT4 measurements would have decreased from 49,858 to 6205 and the projected number of FT3 measurements from 19,899 to 2478. Overall, the total number of thyroid function tests would have decreased from 130,413 to 69,339, representing 61,074 fewer tests (46.8%). According to the predefined testing algorithm, 44,319 FT4 requests (73.1% of all sessions; 88.9% of clinician-requested FT4 tests) and 18,647 FT3 requests (30.7% of all sessions; 93.7% of clinician-requested FT3 tests) were classified as algorithm-defined potential overuse, whereas the projected algorithm would have generated 666 additional FT4 and 1226 additional FT3 measurements.

In the PSA cohort, total PSA concentrations were <4.0 ng/mL in 3539 (77.2%) sessions, 4.0–10.0 ng/mL in 642 (14.0%), and >10.0 ng/mL in 402 (8.8%). Under the predefined testing algorithm, the projected number of free PSA measurements would have decreased from 2133 to 642. Overall, the total number of PSA-related tests would have decreased from 6716 to 5225, corresponding to 1491 fewer tests (22.2%). According to the predefined testing algorithm, 1845 free PSA requests (40.3% of all sessions; 86.5% of clinician-requested free PSA tests) were classified as algorithm-defined potential overuse, whereas the projected algorithm would have generated 354 additional free PSA measurements.

### 3.4. Projected Economic Impact

The projected economic impact of implementing the predefined testing algorithms is presented in Table 4. In the thyroid pathway, projected expenditure decreased from USD 73,579.01 to USD 39,121.06, corresponding to a projected saving of USD 34,457.95 (46.8%). In the PSA pathway, projected expenditure decreased from USD 6468.78 to USD 4560.73, corresponding to a projected saving of USD 1908.05 (29.5%). Overall, implementation of the predefined algorithms was projected to reduce laboratory expenditure from USD 80,047.79 to USD 43,681.79, representing an estimated saving of USD 36,366.00 (45.4%) (Table 4).

## 4. Discussion

In this study, we evaluated the projected impact of applying predefined reflex testing algorithms to real-world clinician-initiated thyroid function and PSA test requests. Comparison of observed ordering patterns with algorithm-based projections identified substantial opportunities to reduce downstream testing and laboratory expenditure. The projected reduction was particularly marked for thyroid function testing, largely reflecting the frequent concurrent ordering of FT4 and FT3 with the initial TSH request, despite most TSH results remaining within the reference interval. A similar, although less pronounced, pattern was observed for PSA testing, where free PSA was frequently requested outside the predefined reflex criteria. Collectively, these findings suggest that structured, algorithm-based laboratory testing pathways have the potential to improve test utilization by reducing downstream testing outside predefined criteria while preserving access to additional testing when those criteria are met.

Our thyroid findings are consistent with recent studies showing that concurrent ordering of TSH with free thyroid hormones remains common and that reflex-based strategies can substantially reduce downstream testing. Murthy et al. reported that 72.5% of thyroid test orders included TSH together with FT4 and/or FT3, while 82–87% of TSH results remained within the reference range. After reinforcement of their reflexive testing algorithm, FT4 and FT3 testing volumes decreased by approximately 60% and 40%, respectively [7]. Similarly, Chan et al. showed that, following implementation of a TSH-first reflex strategy, the proportion of FT4 tests associated with an abnormal TSH increased from approximately 26% to 39%, although simultaneous TSH and FT4 ordering persisted in 19% of tests in 2023 [6]. In a large pediatric hospital system, Warshawsky et al. reduced concurrent TSH–FT4 ordering from 67% to 15% and increased reflex FT4 use from 0% to 77% through a combination of reflex testing, order-entry redesign, clinical decision support, and provider education [11]. These observations are supported by a recent systematic review of 47 studies and 54 interventions, in which 52 interventions reduced low-value thyroid testing and 40 achieved reductions of at least 20%; structural interventions, particularly clinical decision support systems, showed the most consistent benefit [12]. Although our study modelled rather than implemented such an intervention, the magnitude and direction of the projected reduction in thyroid testing are in keeping with the effects reported after real-world stewardship interventions.

Substantial discordance with the predefined testing algorithm was also observed in the PSA pathway, although the projected reduction in overall testing was less pronounced than for thyroid function testing. Previous utilization studies have similarly identified frequent free PSA testing outside the clinical conditions for which the test is most informative. In a national reference-laboratory study, Jackson and Roberts reported that 38% of free PSA tests were performed when total PSA was either below 2.5 ng/mL or above 10.0 ng/mL [9]. A subsequent multicenter Q-Probes study found that predefined testing criteria were met in only 20.6% of free PSA testing episodes [13]. More recently, Çinpolat and Şahin reported that 2522 of 2728 free PSA requests (92.4%) were classified as unnecessary using criteria that included total PSA values below 4 ng/mL or above 10 ng/mL, isolated free PSA requests, and concurrent PSA–free PSA ordering within the intermediate PSA range [14]. Taken together, these studies and our findings indicate that free PSA is frequently requested outside sequential testing pathways and support further evaluation of algorithm-based strategies for guiding its use.

The projected economic impact was also substantial, particularly in the thyroid pathway. This is consistent with prior stewardship studies showing that reductions in downstream thyroid testing can translate into meaningful cost savings despite the relatively low unit cost of individual assays. Murthy et al. estimated savings of approximately Canadian dollars (CAD) 0.65 million in 2024 after reinforcement of a reflexive thyroid testing algorithm, together with marked reductions in FT4 and FT3 utilization [7]. In a pediatric tertiary care hospital, implementation of electronic medical record–based interventions incorporating reflex FT4 testing, indication-based ordering, and decision support reduced FT4 and triiodothyronine (T3) testing by 30% and 66%, respectively, with estimated annual savings of approximately USD 43,000 [15]. Warshawsky et al. similarly reported 5179 fewer FT4 tests over three years, corresponding to direct cost savings of approximately USD 45,800 [11]. Taher et al. reported that implementation of a reflex-based quality-improvement strategy reduced FT4 and FT3 testing and generated estimated savings of CAD 22,018 during the first 27-month intervention cycle [16]. Experience from large integrated healthcare systems further indicates that organized diagnostic stewardship programs can provide both resource optimization and financial benefits while maintaining access to clinically necessary testing [17]. Economic consequences of free PSA testing have also been reported. At a tertiary cancer center, Allard et al. found that 49% of reflex free PSA measurements were clinically unnecessary, corresponding to an estimated annual unnecessary testing cost of CAD 8647.76 [18]. In a modelling study more closely resembling our approach, Caldarelli et al. simulated a reflex algorithm restricting free PSA measurement to total PSA concentrations between 2 and 10 ng/mL and projected a 54.5% reduction in free PSA testing, with reagent savings of €8971 in one year [19]. Although absolute monetary estimates depend on local reimbursement structures and assay pricing, these findings support the broader economic relevance of laboratory stewardship in high-volume testing pathways.

Translation of these projected reductions into clinical practice would require integration of reflex testing with clinical judgment rather than rigid restriction of downstream testing. In practice, TSH or total PSA could serve as the default first-line test, with the laboratory information system automatically initiating the appropriate downstream assay when predefined criteria are met. However, clinicians should retain the ability to request additional testing when specific clinical circumstances justify deviation from the reflex pathway. Such exceptions may be particularly relevant in patients with suspected pituitary dysfunction or established thyroid disease requiring treatment monitoring, for whom additional thyroid hormone measurements may be clinically indicated [5]. Similarly, PSA-related investigations should remain responsive to individual clinical context and urological assessment. Thus, the proposed algorithms should be regarded as default stewardship pathways for routine testing rather than as absolute restrictions on clinician-directed diagnostic evaluation.

This study should be interpreted in light of several methodological considerations. The analysis was based on real-world clinician-initiated laboratory requests and applied the same predefined approach to two commonly used testing pathways. However, this was a retrospective modelling study, and the projected reductions were estimated by applying predefined algorithms to historical laboratory requests rather than by prospectively implementing these algorithms in clinical practice. The projected changes in test utilization and expenditure therefore may not fully reflect those that would be observed after real-world implementation. Prospective implementation studies are therefore needed to establish the clinical effectiveness and safety of these strategies. In addition to test utilization and cost, such studies should evaluate clinician override rates, repeat or subsequent testing, time to diagnosis, and clinically relevant diagnostic outcomes, including potential delayed or missed diagnoses. This would help determine whether reductions in laboratory utilization can be achieved without compromising clinically appropriate testing. In addition, this study was conducted at a single tertiary-care, university-affiliated center, and nearly 90% of testing sessions in both cohorts originated from outpatient care. Ordering patterns and the potential impact of reflex testing may differ in primary care, community hospitals, and other laboratory settings because of differences in patient populations, disease prevalence, referral pathways, and local testing practices. Therefore, the magnitude of the projected reductions observed in this study should not be assumed to apply directly to other healthcare settings, and local validation would be appropriate before implementation. Nevertheless, the underlying stewardship principle of using an initial marker test to guide predefined downstream testing may be transferable across settings when algorithms and reflex thresholds are adapted to local clinical and laboratory practice. Finally, detailed patient-level clinical information was not available for individual test requests. Accordingly, the classification of algorithm-defined potential overuse reflects discordance with the predefined testing pathways rather than a determination that individual tests were clinically unnecessary. Some downstream tests outside these pathways may have been justified by patient-specific factors, including symptoms, previous laboratory abnormalities, known disease, or treatment-monitoring requirements. Therefore, the estimated reductions should be interpreted as the projected effect of applying the predefined algorithms to the observed testing sessions rather than as the number of tests that were definitively unnecessary in clinical practice.

## 5. Conclusions

In conclusion, retrospective application of predefined reflex testing algorithms to clinician-initiated thyroid function and PSA test requests projected substantial reductions in downstream testing and laboratory expenditure, with a greater projected effect in the thyroid pathway. These estimates reflect algorithm-based projections and should not be interpreted as indicating that individual clinician-requested tests were necessarily inappropriate. Prospective studies incorporating patient-specific clinical information and appropriate clinician override mechanisms are needed to determine whether these reductions can be achieved safely in routine clinical practice and across different healthcare settings.

## Figures and Tables

**Table 1:** Characteristics of the study cohorts.

Characteristic	Thyroid Cohort	PSA Cohort
Testing sessions, *n*	60,656	4583
Patients, *n*	43,839	3631
Age, years, median (IQR)	44.0 (25.0–61.0)	66.0 (58.0–72.0)
Outpatient sessions, *n* (%)	54,536 (89.9%)	4194 (91.5%)
Inpatient sessions, *n* (%)	6120 (10.1%)	389 (8.5%)

PSA, prostate-specific antigen; IQR, interquartile range.

**Table 2:** Initial clinician ordering patterns.

Panel A. Thyroid Function Testing	
Initial clinician order	*n* (%)
TSH only	10,756 (17.7)
TSH + FT4	30,001 (49.5)
TSH + FT3	42 (0.1)
TSH + FT4 + FT3	19,857 (32.7)
Total	60,656 (100)
**Panel B. PSA Testing**	
Total PSA only	2450 (53.5)
Total PSA + free PSA	2133 (46.5)
Total	4583 (100)

TSH, thyroid-stimulating hormone; FT4, free thyroxine; FT3, free triiodothyronine; PSA, prostate-specific antigen.

**Table 3:** Observed clinician-initiated testing and projected utilization under the predefined algorithms.

Metric	Thyroid	PSA
**Observed clinician-initiated downstream tests, n**		
FT4/Free PSA	49,858	2133
FT3	19,899	—
**Projected algorithm-based downstream tests, n**		
FT4/Free PSA	6205	642
FT3	2478	—
**Algorithm-defined potential overuse**		
FT4/Free PSA, *n* (%)	44,319 (73.1)	1845 (40.3)
FT3, *n* (%)	18,647 (30.7)	—
**Projected additional testing**		
FT4/Free PSA, *n* (%)	666 (1.1)	354 (7.7)
FT3, *n* (%)	1226 (2.0)	—
**Total observed tests**	130,413	6716
**Total projected tests**	69,339	5225
**Net reduction, *n* (%)**	61,074 (46.8)	1491 (22.2)

PSA, prostate-specific antigen; FT4, free thyroxine; FT3, free triiodothyronine.

**Table 4:** Projected economic impact of the predefined algorithms.

Cost Measure	Thyroid Pathway (USD)	PSA Pathway (USD)	Overall (USD)
Observed clinician-initiated cost	73,579.01	6468.78	80,047.79
Projected algorithm-based cost	39,121.06	4560.73	43,681.79
Projected saving	34,457.95	1908.05	36,366.00
Projected reduction (%)	46.8	29.5	45.4

USD, US dollars.

## Data Availability

The datasets generated and/or analyzed during the current study are available from the corresponding author on reasonable request.

## Abbreviations

The following abbreviations are used in this manuscript:

PSA	prostate-specific antigen
TSH	thyroid-stimulating hormone
FT4	free thyroxine
FT3	free triiodothyronine
